# Solid-State Nanopore

**DOI:** 10.1186/s11671-018-2463-z

**Published:** 2018-02-20

**Authors:** Zhishan Yuan, Chengyong Wang, Xin Yi, Zhonghua Ni, Yunfei Chen, Tie Li

**Affiliations:** 1 School of Electromechanical Engineering, Guangdong University of Technology, Guangzhou, 510006 China; 2 School of Medicine, South China University of Technology, Guangzhou, 510006 China; 3 Jiangsu Key Laboratory for Design and Manufacture of Micro-Nano Biomedical Instruments, Southeast University, Nanjing, 211189 China; 4 Science and Technology on Microsystem Laboratory, Shanghai Institute of Microsystem and Information Technology, Chinese Academy of Sciences, Shanghai, 200050 China

**Keywords:** Solid-state nanopore, Fabrication, Applications

## Abstract

Solid-state nanopore has captured the attention of many researchers due to its characteristic of nanoscale. Now, different fabrication methods have been reported, which can be summarized into two broad categories: “top-down” etching technology and “bottom-up” shrinkage technology. Ion track etching method, mask etching method chemical solution etching method, and high-energy particle etching and shrinkage method are exhibited in this report. Besides, we also discussed applications of solid-state nanopore fabrication technology in DNA sequencing, protein detection, and energy conversion.

## Background

Solid-state nanopore has attracted increasing attention, due to adjustable size, high reliability, easy to modify, and so on [1–3]. It has been applied to DNA sequencing [4], water purification [5], protein detection [6], nanoparticle separation [7], energy conversion [8], and so on, especially in the area of DNA sequencing, protein detection, and energy conversion. So, it is very significant to fabricate solid-state nanopore with low-cost and high-efficiency method.

Solid-state nanopore fabrication technology was first reported by Jiali Li and her collaborator in 2001 [9] and has become a hot spot of research. According to the manufacturing mechanism, solid-state nanopore fabrication technology can be summarized into two broad categories. The first one is “top-down” etching technology, such as focused ion beam and high-energy electron beam. The second type is “bottom-up” shrinkage technology, which was based on the first type, such as electron beam-assisted deposition and atomic layer deposition. Now, silicon nitride [10] and silicon oxide [6] have been used to prepare solid-state nanopore, which possessed excellent performance such as adjustable diameter and length of channel. Besides, graphene [11] and molybdenum sulfide [12] can also be used to fabricate solid-state nanopore.

The diameter of the solid-state nanopore can be precisely controlled from subnanometer to several hundred nanometers according to the need [13]. In general, solid-state nanopore is prepared on insulating materials [14] and is very stable in extreme solutions such as concentrated sulfuric acid [15] and high temperatures [16]. However, their stability is also largely dependent on the method of preparation. In this paper, we review the preparation method of solid-state nanopore. Firstly, we have discussed the development of solid-state nanopore fabrication technology. Then, we exhibit various solid-state nanopore fabrication technologies in detail. Finally, we summarized applications of solid-state nanopore fabrication technology in some area.

## Development Process

**Fig. 1:**
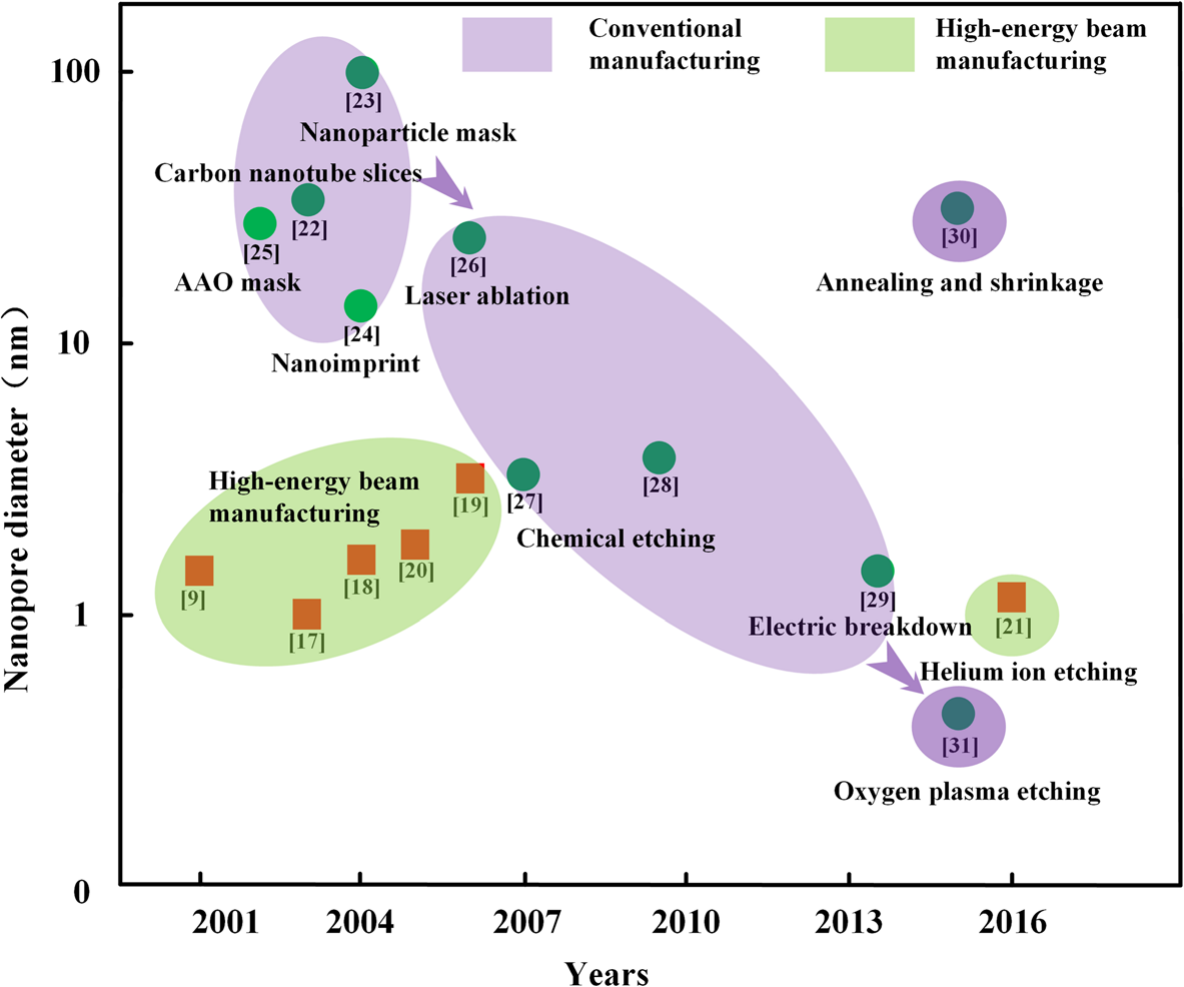
Solid-state nanopore manufacturing technology development roadmap

Since Jiali Li of Harvard University first reported the production of silicon nitride nanopore by argon ions in 2001 [9], the solid-state nanopore fabrication technology gradually developed into two branches of high-energy beam manufacturing [17–19] and conventional manufacturing (Fig. 1). Researchers try to improve the efficiency of solid-state nanopore manufacturing with high-energy beam to make up for the lack of high cost. Gierak et al. [20] improved the Ga^+^ direct writing system of the focused ion beam (FIB) and produced a nanopore on a 20-nm-thick SiC film with the diameter of about 2.5 nm. In 2016, helium ion etching system with high efficiency appeared, and it possessed smaller active region of beam spot and sample. Until now, it has processed Si_3_N_4_ nanopore with the diameter of only 1.3 nm [21].

It has always been the pursued goal by researchers to achieve efficient and controllable fabrication of solid-state nanopore using conventional manufacturing methods. Due to the demand of solid-state nanopore, there appear many solid-state nanopore fabrication technologies, such as carbon nanotube dicing [22], mask etching (nanosphere [23] and porous anodic alumina film [24]), nanoimprint [25], and so on. Although these methods avoid the use of transmission electron microscope (TEM), FIB, and other expensive processing equipment, there are still many deficiencies. The controllability of carbon nanotube cutting method is poor, which is not suitable for batch manufacturing. Nanosphere diameter in mask etching limits the size and density of triangular solid-state nanopore. Porous anodic aluminum oxide films have low strength and require the assistance of transfer process, which reduce manufacturing efficiency. Nanoimprint requires the high-precision templates, which in itself is a micro/nanomanufacturing challenge.

After Ling et al. fabricated the plastic nanopore by current feedback control technology, this technology has been used for silicon etching [26], and the controllable fabrication of silicon nanopore was realized [27]. Based on the work of Ling, Pedone et al. [28] used electron beam lithography to fabricate silicon-etched windows, which improved the orifices differences caused by photolithographic errors. Later, the researchers combined current feedback control technology with electrical breakdown technology and created solid-state nanopore below 2 nm [29]. However, the current feedback control technique cannot identify the increased current signal caused by whether the increase of the pore number or the increase of the single-pore diameter. So, it is not suitable for the fabrication of solid-state nanopore.

Recently, Liu et al. [30] fabricated nanofluid field effect tube based on glass pores using micrometer cell etching, glass deposition, and annealing and atomic layer deposition methods. Surwade et al. [31] used oxygen plasma etching on graphene and obtained graphene nanopore film with a diameter of 0.5–1 nm. Although the material of this nanoporous manufacturing technology is limited to graphene, and the transfer process of graphene is not compatible with micro-electro-mechanical system (MEMS) and complementary metal oxide semiconductor (CMOS) process, its mechanism of making pores has broken the minimum surface energy limit, which prove the coming of solid-state nanopore manufacturing with high efficiency and low cost.

## Fabrication Technologies

### Ion Track Etching Method

Solid-state nanopore first was fabricated with ion track etching. Ion track etching used etchant to etch the film, which was irradiated by heavy ion. The etching rate of the track region is greater than that of the non-track region (*v*_track_ > *v*_bulk_), which result in the form of pore. This method has successfully fabricated solid-state nanopore in relatively inexpensive materials such as polycarbonate, polyimide, and silicon nitride. Zhang et al. [32] has fabricated silicon nitride nanopore by this method with high-energy Br^+^ (81 MeV). The diameter of this nanopore was relatively large, and the minimum nanopore diameter obtained was 40 nm after the process of shrinkage. At present, Harrell et al. [18] have fabricated the solid-state nanopore with the diameter of 2 nm by ion track etching, after the diameter was shrunk by the deposition of nanogold thin films. However, the solid-state nanopore prepared by the ion-channel etching method has a small porosity and an uneven pore size distribution. Meanwhile, this method requires expensive heavy ion accelerometer and restricts the fabrication and application of the solid-state nanopore severely.

### Mask Etching Method

**Fig. 2:**
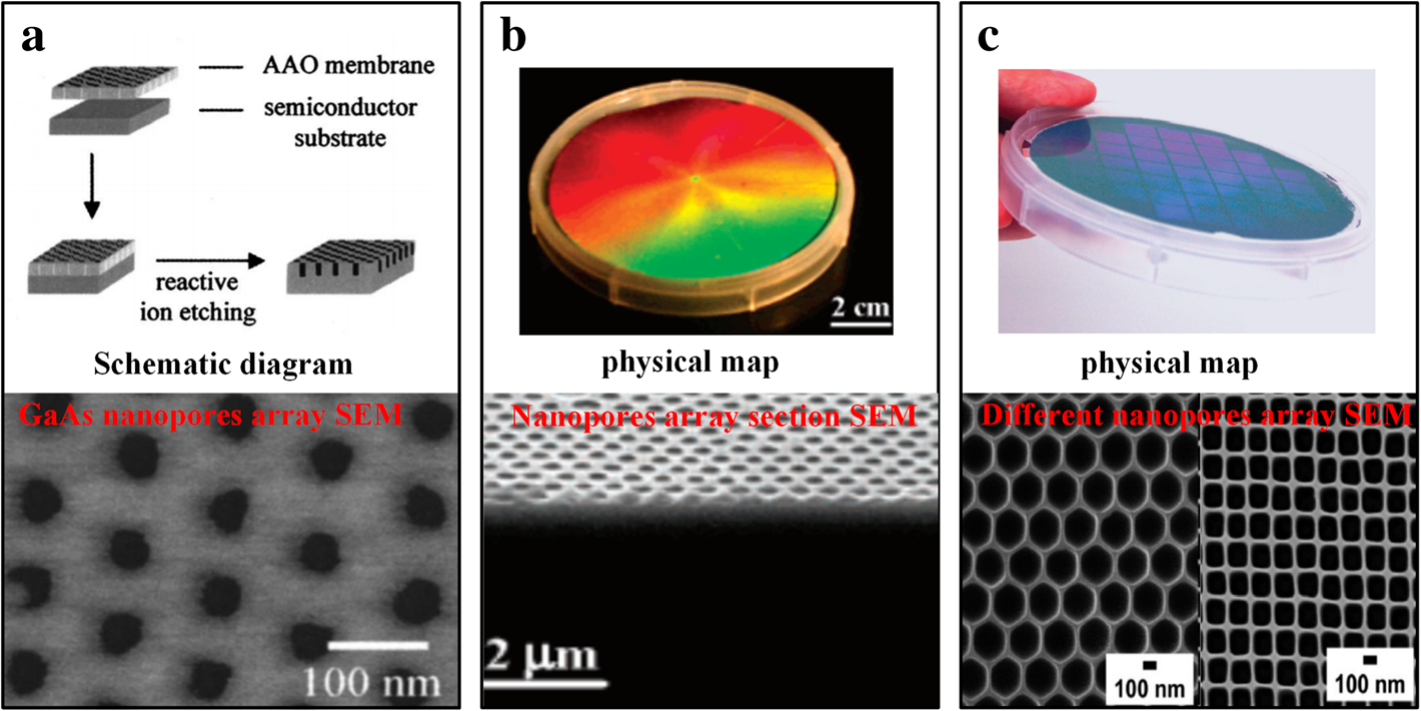
Preparation of solid-state nanopore by mask etching methods. (**a**) GaAs solid-state nanopore [25], (**b**) silicon nanopore [33], and (**c**) aluminum nanopore with different shapes [34]

Mask etching method can be divided into three auxiliary manufacturing methods according to the type of mask, which was porous anodic aluminum oxide (AAO), nanosphere, and nanoimprint respectively. Researchers found that the AAO not only possess uniform pore size distribution and adjustable pore length but also has periodic honeycomb pore structure without cross and connection between the pore in the side. It can overcome the problem of low porosity and uneven size distribution in the ion track etching method. As shown in Fig. 2a, Liang et al. [25] have transferred the nanopore pattern onto the substrate by reactive ion etching using AAO as a mask and realized controlled fabrication of the solid-state nanopore. Unfortunately, the mechanical strength of AAO film is poor, and it is prone to cracking. Besides, its manufacturing process also exists many problems, such as time consuming, low production, polluting environment, and wasting of raw materials. These defects all limit the use of AAO mask etching methods.

Inspired by AAO mask etching to fabricate solid-state nanopore, Alyson et al. [24] use the nanosphere as mask, followed by a reactive ion etching (RIE) to create a high-porosity solid-state nanopore with a triangular cross section. Chen et al. [33] based on the former and adjusted the diameter of the nanospheres of upper layer in double-layer polystyrene nanospheres to control the gap distribution and size of nanosphere precisely. Finally, by means of deep reactive ion etching, they obtained a silicon nanopore with a depth up to 2 μm whose cross section was similar to that of the nanosphere. Nanosphere etching technology also can be combined with metal deposition or stripping process to produce a metal nanopore mask. Then, combined with etching and removing the metal mask process, a silicon nanopore was obtained [34] (Fig. 2b). Nanosphere etching technology possesses wide adaptability, which not only can be used to create solid-state nanopore with multi-layer structure but also can be used to create high-porosity polyethersulfone filter. However, due to the diameter limitation of the nanospheres, the diameter of nanopore is too large, and it is difficult to less than 10 nm.

It is very complex to fabricate solid-state nanopore by AAO masks or nanosphere auxiliary manufacturing methods because it involve in the mask fabrication, transfer, and removal processes. At the same time, the mask cannot be reused and results in waste. So, researchers have turned their attention to reusable nanoimprint technology. The principle of nanoimprint is to press a prepared template onto a thin polymer film (such as polymethyl methacrylate), and the pattern, which is similar to the template, is obtained when the film is solidified [35]. Nanoimprint technology not only can reuse the templates but also can produce complex nanostructures with a minimum line width up to 5 nm [23]. Porous aluminum is the most common product with nanoporous structure fabricated by nanoimprint technology [36] (Fig. 2c). Currently, Chou et al. [37] have created the smallest nanopore by nanoimprint technology. They used chromium as a mask and used electron beam etching and RIE obtaining a diameter of 10 nm and a height of 60-nm SiO_2_ nanopillar. Subsequently, the diameter of the nanopillar is further reduced by HF etching, and nanopore with the diameter of less than 6 nm is obtained by using the nanopillar as an imprint template. However, the stability of this method is poor, and the template manufacturing and stamping process still need improvement. The high-precision templates are required in nanoimprint technology and need nanoscale manufacturing methods such as electron beam lithography to manufacture, which in itself is a challenge in micro/nanofabrication. In addition, the life of the template and imprint precision also are the challenges of nanoimprint technology.

### Chemical Solution Etching Method

**Fig. 3:**
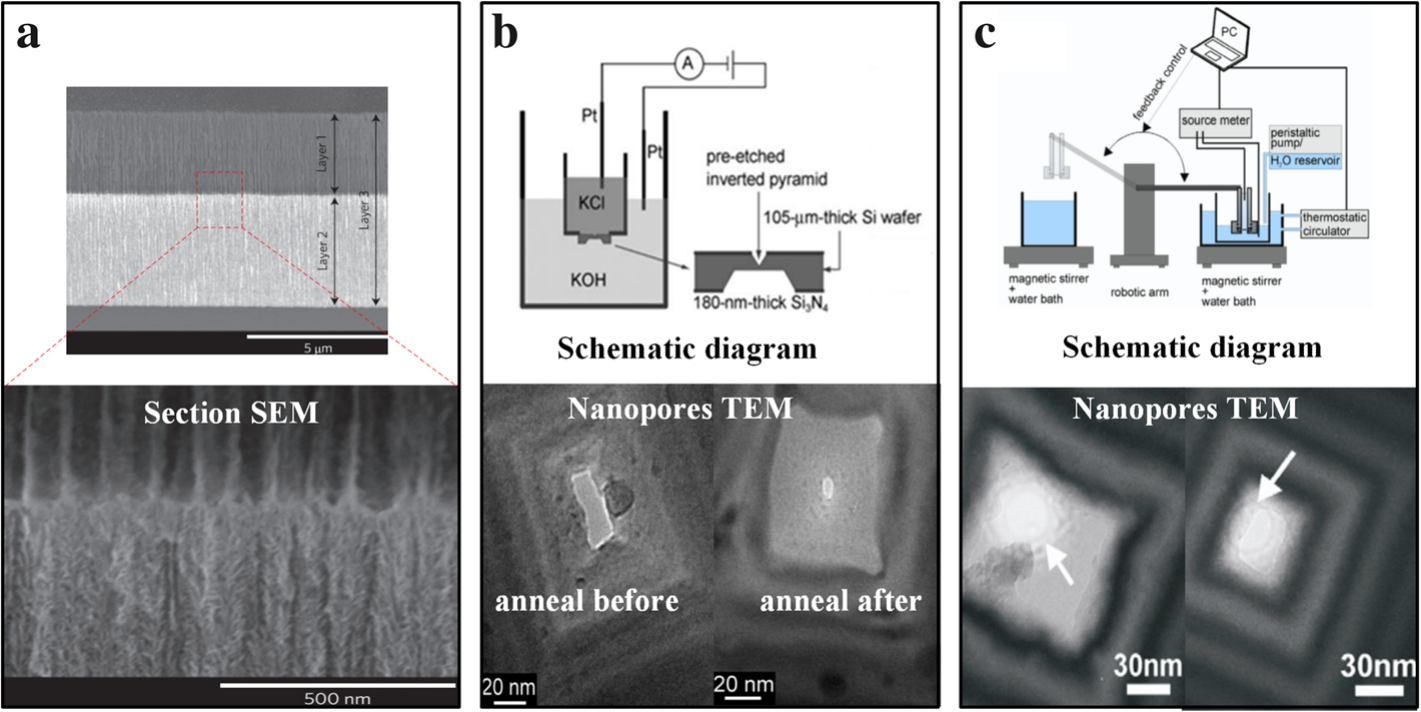
Preparation of solid-state nanopore by chemical solution etching. (**a**) Double-walled silicon nanopore [38], (**b**) silicon nanopore [27], and (**c**) highly controllable silicon nanopore [28]

In addition to using mask etching methods, scientists are also trying to fabricate solid-state nanopore using chemical solution etching. Among chemical solution etching, electrochemical etching methods are commonly used in the manufacture of porous silicon. Electrochemical etching method is a cheap method for manufacturing silicon solid-state nanopore and can precisely control the pattern and location of porous silicon by designing the mask. In addition, the porosity and nanopore size of porous silicon can also be controlled by adjusting the etching liquid concentration, etching current, etching time, and other process parameters. Orosco et al. [38] have obtained outstanding achievement by this method and have produced double layers of porous silicon with minimum nanopore diameter of 6 nm (Fig. 3a). In addition, Wang et al. [39] used a focal ion beam (dose of 10^11^~10^15^ ions/cm^2^) to irradiate the specific position of silicon, then electrochemical etching method was used to obtain the silicon nanopore with controlled position and quantity, while the number and size of nanopore all are limited by the small view field of the ion beam. However, the surface roughness of porous silicon wall fabricated by electrochemical etching method was too high even existing bifurcation structure, which seriously restricts the application of electrochemical etching method used to fabricate silicon solid-state nanopore.

With the development of MEMS technology, researchers have found that the chemical solution etching technique can be used to fabricate silicon nanopore with controlled positions and numbers [27, 28, 40]. Park et al. [27] firstly used the solid-state nanopore fabricated by chemical solution etching technology for DNA sequencing. Firstly, they used photolithography and RIE to etch silicon nitride films on both sides of the silicon wafer and obtain silicon windows with different areas on. Next, the silicon wafer is placed in KOH solution for etching, and an inverted pyramid and trapezoidal structure were obtained in small and large windows respectively. Thirdly, the silicon wafer is mounted on the feedback etching system, and the KCl salt solution and the KOH etching solution are isolated by a silicon wafer (Fig. 3b). When the KOH solution pierces the silicon wafer obtaining the nanopore, the solution on both sides of the silicon wafer passes through the nanopore and conducts the Pt electrodes obtaining a feedback electrical signal. Finally, they remove the silicon wafer obtaining silicon nanopore. Due to the limitations of lithography mask fabrication and photolithographic errors, the small patterned silicon window cannot be an absolute square, so the etched solid-state nanopores are approximate rectangles and require subsequent processing such as annealing to improve the morphology of the pores. Pedone et al. [28] developed a small window using electron beam lithography based on the former, which avoided the mask manufacturing and lithography error. At the same time, when the electrical signal feedback was added in the intelligent control system, the approximately perfect nanopore was obtained (Fig. 3c). In similar way, Liu et al. [41] used a combination of dry and wet etching methods to fabricate silicon nanopore with the minimum diameter of 30 nm. Not difficult to find, in addition to Rant groups, other groups just can fabricate silicon nanopore with larger diameter. At the same time, it is difficult to characterize the diameter of the nanopore, which attributes the limited field of TEM.

### High-Energy Particle Etching and Shrinkage Method

After encountering setback in the quest to fabricate solid-state nanopore using simple methods, some researchers returned to using energetic particles to fabricate nanopore in small areas with controllable structure [20, 42]. Kim et al. [42] firstly used focused ion beam etching and obtained 6 × 6 blind pore with the diameter of 2 μm as an electron beam lithography area. Then, they used high-energy electron beam etching in TEM obtaining the SiN nanopore, and the average diameter of the resulting SiN nanopore was 5.14 nm with a standard deviation of 0.46 nm. Due to the limitations of the TEM equipment, only one chip can be placed in each vacuum, which severely restricts the fabrication rate of the nanopore chip. FIB device possesses larger cavity, and it can be placed more than one chip even a whole wafer (silicon). Comparing with TEM, it has greatly increased the manufacturing efficiency of nanopore. However, the diameter of nanopore fabricated by focused ion beam etching is too large. At present, only Gierak group have fabricated nanopore with diameters less than 5 nm using FIB [20]. They improved the Ga^+^ direct writing system and fabricated nanopore with diameter about 2.5 nm on silicon carbide film with the thickness of 20 nm.

Now, apart from Gierak groups, it is difficult for other groups to use the Ga^+^ source focused ion beam system to fabricate nanopore with diameter less than 10 nm. The researchers try to use FIB to make larger diameter nanopore, then surface treatment was used to reduce the diameter of the nanopore [43–46]. So far, methods for reducing nanopore diameter have been divided into two categories. The first type is the deposition means, in which material was deposited in the nanopore surface to reduce the diameter of the nanopore. The second type is the electron beam irradiation, which make the material of nanopore edge migrate and reduce the nanopore diameter.

#### Nanopore Surface Deposition Material Shrinkage

**Fig. 4:**
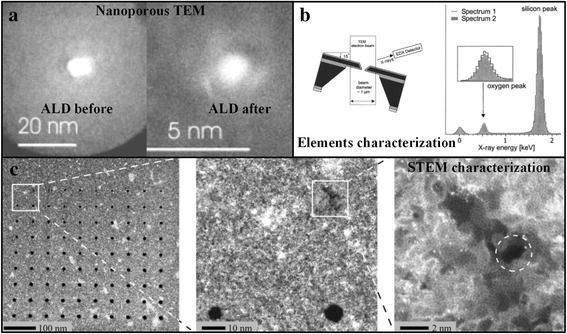
High-energy particle etching and modification methods for the fabrication of solid-state nanopore. (**a**) ALD shrinkage, (**b**) self-calibration of nanopore edge, and (**c**) helium ion etching nanopore

Chen et al. [43] firstly realized precise reduction of nanopore diameter by depositing materials on the nanopore surface. They deposited 24 layers of alumina on the Ga^+^-etched nanopore surface using atomic layer deposition (ALD), and the nanopore diameter was reduced to 2 nm (Fig. 4a). During DNA sequencing process, it was found that the nanopore prepared by this method can effectively reduce the noise and improve the signal-to-noise ratio. The essence of atomic layer deposition method is the sub-nanometer single-layer deposition process, and it possesses stable process which is beneficial for the precise manufacture of nanopore. Torre et al. [44] employed similar approach to reduce nanopore diameter, in which they firstly used focused ion beam etching to obtain nanopore with an average diameter of 27.3 nm, then nanopore diameter was reduced to 8.3 nm by deposition of titanium oxide using ALD.

Rant et al. found another way. They firstly used electron beam lithography and RIE to obtain silicon nitride nanopore. Then, the nanopore was reduced to below 10 nm by depositing Ti/Au thin film on the nanopore surface using a physical evaporation method [45]. In addition to alumina, titanium oxide, and metal, amorphous carbon also can be deposited for shrinkage with the assistance of electron beam in FIB system [46].

#### Nanopore Edge Material Migration Shrinkage

The migration of nanopore edge material is based on the principle of the nanopore surface energy minimum, which was proposed by Dekker group [47]. That is, when the nanopore diameter is smaller than the nanopore thickness, nanopore will be shrunk irradiated by high-energy electron beam. Based on the research of Dekker, Storm et al. [48] in situ observed that the minimum diameter of the silicon oxide nanopore was shrunk to 2 nm after irradiated by the electron beam (Fig. 4b). This experimental result further confirmed nanopore surface energy minimum principle. In addition, the energy dispersive X-ray spectroscopy (EDX) and electron energy loss spectroscopy (EELS) also confirm that the decrease of nanopore diameter was resulted from the migration of the nanopore edge material, instead of caused by nanopore surface contamination [9]. The principle of nanopore surface energy minimum is verified in different morphologies of silicon oxide nanopore, such as elliptical silicon oxide nanopore and silicon nitride/silica composite nanopore [49].

The shrinkage method solves the problem that the size of the nanopore in the FIB fabrication is not small enough, but the manufacturing process of the nanopore is complicated. Researchers have also been pursuing simpler ion beam fabrication methods to fabricate solid-state nanopore. Recently, the emergence of nanopore fabrication technology with helium ion etching, which possesses smaller active area of beam spot and sample, overcomes the difficulty of conventional FIB, in which the diameter of nanopore is larger than 10 nm. The Emmrich et al. [21] have demonstrated that this system can produce silicon nitride nanopore with diameter of only 1.3 nm and thickness of 30 nm (Fig. 4c). Although it has greatly increased processing efficiency comparing with TEM and focused ion beam systems using conventional Ga^+^ ion sources, this system is expensive which limits the application of it.

### Electrochemically Confined Nanopore Method

Ying et al. and Lin et al. [50, 51] initiate the concept of electrochemically confined nanopore which exhibits the excellent capability to ingeniously confine the electrochemistry, energy distribution, optical enhancement, and the mass transport within the asymmetric nanopore. Confined nanopore electrode (CNE) can be used to perform high-resolution time-resolved studies of electrochemical processes within a single cell by using nanoparticle-confined nanoparticulate electrodes in normal chemical laboratories. With the help of optics, it can also be applied to multi-dimensional simultaneous acquisition of single-body photoelectric signals at the nanoscale, providing new ideas for the electrochemical measurement of single living cells, single particles, and single molecules [52].

## Application

### DNA Sequencing

After the idea of nanopore, DNA sequencing was put forward by the biologist Kasianowicz group in 1996 [53]; the nanopore technology has been rapidly developed. DNA sequencing using nanopore is a physical method, and it replaced Sanger’s DNA polymerase method. This method uses the electric field to drive the movement of DNA in the nanopore, and it directly uses the time characteristic of nanopore ion current to distinguish the size of a single base so as to achieve the purpose of DNA sequencing. Nanopore DNA sequencing method avoids DNA modification, amplification, and other processes, which save the cost of expensive polymerase, so this method possessed high competitiveness. Inspired by Kasianowicz, physicists began investigating the possibility of this method since 2000, so the field of nanopore DNA sequencing was born.

Nanopore DNA sequencing method can be divided into bio-nanopore sequencing and solid-state nanopore sequencing according to the nanoporous material [54]. Among them, bio-nanopore sequencing exists the disadvantages of DNA molecules’ pause and reverse, which makes the current-time signal detected by this method misinterpreted [55]. As a result, solid-state nanopore DNA sequencing and its fabrication have become the hot topics of scholars in various countries [56].

**Fig. 5:**
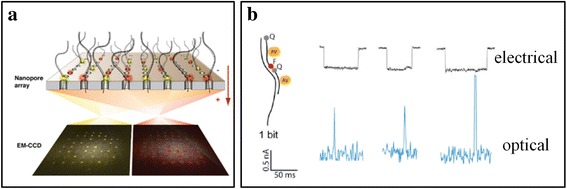
Total internal reflection fluorescence (FTIR) parallel detection of DNA sequence [58]. **a** Schematic diagram. **b** Signal map of optical and ion current signals detected in the experiment

With the deep research of nanopore DNA sequencing methods, scientists think that nanopore sensors can realize the parallel detection of DNA and achieve the goal of high-throughput DNA sequencing [57]. One of the most promising is the fluorescence parallel detection of DNA sequence technology, which was based on solid-state nanopore internal reflection [58] (Fig. 5). With the help of electron multiplying charge-coupled device (CCD) camera, it can be captured of the DNA via signal of each nanopore, and multiple optical signals and ion-current signals can be corresponded one to one to realize high-throughput DNA sequencing. Subsequently, this technology was further confirmed by bio-nanopore sequencing, which theoretically allowed the identification of 10^6^ base/mm^2^ per second [59]. However, there are also some disadvantages for the solid-state nanopore DNA sequencing methods, such as the high translocation speed and the low spatial resolution [60].

### Protein Detection

In 2007, Fologea et al. [61] successfully detected bovine serum albumin (BSA) using solid-state nanopore with thickness of 10 nm. Besides, they also studied the conformation change of β-lactoglobulin under the action of different concentrations of urea denaturant by solid-state nanopore. They found that most of the proteins pass through the nanopore with linear or helical conformation and that the electric field in the nanopore could unwind the passing protein [62]. So, they started out the detection of proteins and the research of physicochemical properties and structure of proteins. Cressiot et al. [63] fabricated solid-state nanopore with the diameter of 20 nm using FIB and systematically studied and compared the characteristics of current signals when wild-type maltose binding protein (MaIE) and unfolded MaIE passed through the nanopore. In this experiment, they also found that there was a free energy barrier when the protein passed through the nanopore. After that, Cressiot fabricated the nanopore with the diameter of 3 nm using TEM and found the MaIE protein again. In contrast, the protein was stretched by the electric field when the electric field was large.

In 2013, Plesa et al. [64] successfully tested aprotinin (6.5 kDa), ovalbumin (6.5 kDa), beta-amylase (45 kDa), ferritin (200 kDa), and thyroglobulin (660 kDa); five proteins using silicon nitride nanopore with the diameter of 40 nm. They found that the measured current signal was distortion because the speed was too fast of protein through the nanopore, and the detection bandwidth was relatively small. Besides, the frequency of the event was opposite to the diffusion constant of protein. There are two ways to solve this contradiction. One way is to reduce the speed of protein through nanopore, and the other way is to increase the detection bandwidth. Di et al. [65] successfully reduced the speed of ubiquitin protein through nanopore using low-power visible light and distinguish the rotation angle during the protein through the nanopore. Recently, they successfully detected the ubiquitin protein and distinguish the connection type between ubiquitin protein and protein using solid-state nanopore with the diameter of 3 nm. This work opens up a new avenue for biomedical research of ubiquitin protein [66]. In 2014, Larkin et al. [67] successfully detected proteinase K and RNA enzyme A using high-bandwidth current amplifier and ultra-thin HfO_2_ nanopore and measured the electromobility, diffusion constant, and volume of this protein.

Nanopore possesses extremely high detection resolution for the molecule internal structure, and it has become a powerful sensor for the interaction of single molecule. It has been widely used in real-time detection of DNA-protein interactions, protein-protein interactions, and chemical small molecules. As a result, a series of techniques based on nanopore sensing technology have been produced, such as detection and diagnosis of diseases and detection of heavy metal ions and viruses.

### Energy Conversion

The development of advanced micron/nanomanufacturing technology provides the basis for the miniaturization and miniaturization of traditional energy conversion device [40, 41]. Many micrometers’ degree of energy conversion devices continuously appears, such as microreactors [42], micro gas turbines [43, 44], micro thermal engines [45, 46], micro fuel cells [47], and micro supercapacitors [48]. Compared with the traditional large-scale energy conversion devices, these miniature energy conversion devices can provide higher energy density. These micro-devices cannot be applied to large-scale energy equipment, due to the high costs of micro/nanoprocessing. However, the characteristic of microminiaturization makes them suitable for the construction of electrical source components with small-scale and low-power consumption to drive electronic equipment, such as nanomachines, micro-electromechanical system, and biomedical implant devices.

Energy conversion method based on nanopore channel takes full advantage of the unique physical-chemical properties of nanoscale. It converts the clean energy existing in environment, such as mechanical energy, chemical energy, light energy, and electric energy. At the same time, it does not emit carbon dioxide, produce vibrations and working noise harmful to the human body, and is very friendly to environment during conversion process. Daiguji et al. [68] converted the mechanical energy to electric energy by solid nanopore channel. Wen et al. [69] converted solar energy to electric energy based on smart-gating nanopore channels. Guo et al. [70] converted salinity gradient energy to electric energy with single-ion-selective nanopore. Table 1 shows several micro-scale energy conversion devices [71].

**Table 1:** Micro-scale energy conversion devices [71]

Energy conversion devices	Energy input	Energy output	Conversion efficiency	Reference
Micro-heat engine	Thermal energy	Electric energy	~ 30%	[75]
Micro-fuel cell	Chemical energy	Electric energy	> 60%	[76]
Photovoltaic device	Luminous energy	Electric energy	~ 12%	[77]
Nanomobile battery	Mechanical energy	Electric energy	~ 40%	[78]
Nanoconcentration battery	Chemical energy	Electric energy	15%~40%	[70]

Energy conversion based on solid-state nanopores was inspired by the research on the function of ion channels of cell membrane [71]. Due to the excellent performance of solid-state nanopores, such as chemical durability, thermostability, superior mechanical property, tunable size and shape and so on [72], it has got increasing attention in the area of energy conversion. For example, Wen et al [73] reported that the nanofluidic energy conversion systems based on solid-state nanopores exhibited high power density, long operating life and good safety performance, compared with other commercially available cation exchange membranes. Besides, along with the development of fundamental studies and practical applications, solid-state nanopores with smart ion transport behaviors, such as ionic selectivity, ionic gating and ionic rectification, has been used as extraordinary platforms for energy conversion [74].

## Conclusions

This report reviews briefly the development process, fabrication technologies, and application of solid-state nanopore. Since Jiali Li firstly reported the fabrication of solid-state nanopore, researchers has always been pursued efficient and controllable manufacturing methods to fabricate solid-state nanopore. A comprehensive analysis of the latest research results on the fabrication of solid-state nanopore shows that the current research are all based on nanometer-scale processing tools, which cannot be mass produced at low cost and high efficiency. Therefore, it is of great significance to study the new method of fabricating solid-state nanopore. Along with the development of the manufacturing methods of solid-state nanopore, it has been applied in various areas, especially in DNA sequencing, protein detection, and energy conversion. In brief, the fabrication and application of solid-state nanopore are a promising area, and it is significant to our economics and living quality. Along with the development of advanced micro/nanomanufacturing technology and new theory, solid-state nanopore will be fabricated with lower cost and higher efficiency, and the application will be wider.

## Abbreviations

AAO: Anodic aluminum oxide
ALD: Atomic layer deposition
CCD: Charge-coupled device
CMOS: Complementary metal oxide semiconductor
EDX: Energy dispersive X-ray spectroscopy
EELS: Electron energy loss spectroscopy
FIB: Focused ion beam
MaIE: Maltose binding-protein
MEMS: Micro-electro-mechanical system
RIE: Reactive ion etching
TEM: Transmission electron microscope
